# Exploring Rafe’s Sciatica: Investigating the Link Between Spondyloarthropathy and Non-discogenic Sciatica

**DOI:** 10.7759/cureus.92405

**Published:** 2025-09-15

**Authors:** Md. Muhibbur Rahman, Nadia Ferdous, Md Mahdi Hasan, Shahina Sarker, Shamim Farhad

**Affiliations:** 1 Physical Medicine and Rehabilitation, Government Employees Hospital, Dhaka, BGD; 2 Medicine, Government Employees Hospital, Dhaka, BGD; 3 Pharmacy, Manarat International University, Dhaka, BGD; 4 Physical Medicine and Rehabilitation, Savar Upazila Health Complex, Savar, BGD

**Keywords:** amor criteria, assessment of spondyloarthritis international society (asas), inflammatory back pain (ibp), piriformis syndrome (ps), sacroiliitis, sciatica, spondyloarthropathy (spa)

## Abstract

Background and aim

Sciatica is commonly attributed to nerve root compression due to disc herniation, foraminal stenosis, or spondylolisthesis, which are well-recognized causes of the condition. However, non-discogenic causes, such as inflammatory disorders, including axial spondyloarthritis, remain underrecognized and contribute significantly to the pathophysiology of sciatica. "Rafe’s sciatica," proposing a distinct subtype of sciatica linked to spondyloarthritis (SpA), is one such example. Other types of sciatica share clinical characteristics with this condition, such as piriformis syndrome, which can also involve sacroiliitis and sciatic nerve entrapment. This study aimed to identify and characterize Rafe’s sciatica as a novel, non-discogenic sciatica subtype associated with SpA, and to explore its diagnostic and clinical implications.

Methods

A prospective case series of 41 patients with sciatica and suspected SpA was conducted at the Government Employees' Hospital in Dhaka, Bangladesh, from November 2022 to July 2023. The purposive sampling technique was applied to recruit patients who met the inclusion criteria. No blinding was applied. Data were collected through structured interviews and medical records. The Assessment of Spondyloarthritis International Society (ASAS) and Amor criteria guided SpA diagnosis. Enthesitis was assessed clinically and via ultrasound. Sacroiliitis was diagnosed using X-ray and magnetic resonance imaging findings, including bone marrow edema, synovitis, and subchondral sclerosis. Metabolic causes were excluded biochemically. Ethical approval for the study was obtained from the Institutional Review Board of the Government Employees’ Hospital, and informed consent was obtained from all participants prior to inclusion.

Results

The average age of the patients was 39 years, with a predominance of females (31, 75.6%). Imaging studies revealed that 35 (85.4%) patients had sacroiliitis, a key feature of SpA, and 12 (29.3%) patients tested positive for the human leucocyte antigen (HLA)-B27 genetic marker, which is commonly associated with inflammatory disorders. Clinically, 35 (85.4%) patients reported persistent low back pain and exhibited sacroiliac joint tenderness, while morning stiffness lasting more than 30 minutes was observed in 28 (68.3%) patients. Additionally, dactylitis was present in 21 (51.2%) patients. Almost all patients had positive results from the FAIR (flexion, adduction, and internal rotation), modified FAIR, and piriformis stretch tests, which further indicated a link to SpA.

Conclusion

Rafe’s sciatica appears to represent a novel, non-discogenic sciatica subtype associated with SpA. Recognition of this condition may improve diagnostic accuracy and patient management by emphasizing the role of clinical markers and imaging in identifying inflammatory causes of sciatica. While our findings highlight its potential clinical relevance, further validation through larger, comparative studies is essential to refine diagnostic criteria and develop effective treatment strategies.

## Introduction

In clinical practice, sciatica is a commonly encountered condition resulting from nerve root compression. Its typical causes include intervertebral disc herniation, lateral or foraminal spinal stenosis, spondylolisthesis, or, in rare cases, spinal tumors [1]. While these causes are well-documented and commonly understood, the diagnosis of other conditions presenting with similar symptoms, such as sacroiliac (SI) joint-related pain and piriformis syndrome (PS), remains significantly more complex. Diagnosing SI joint-related pain during a physical examination is difficult due to the absence of a single reliable diagnostic test. The validity and consistency of different physical tests vary widely, and some can yield positive results in up to 20% of asymptomatic individuals [2]. In practice, diagnosis often depends on recognizing a pattern of clinical findings rather than a definitive test [3]. Although CT and MRI scans can be useful in detecting sacroiliitis and joint space narrowing, no clear radiological marker exists for routine SI joint pain [4]. In this context, intra-articular SI joint injection remains the most definitive diagnostic method to confirm SI joint pathology [5]. PS is a rare cause of sciatic nerve compression, typically due to entrapment or irritation of the sciatic nerve by the piriformis muscle. It often presents with buttock, hip, or posterior thigh pain, sometimes radiating distally in a sciatica-like distribution [6,7]. PS remains underdiagnosed and is often misunderstood, in part due to overlapping symptoms with more common conditions like lumbar disc herniation and SI dysfunction.

Despite the lack of a universally accepted diagnostic protocol, some clinical signs remain relevant. Robinson (1947) proposed six diagnostic features of PS, including a history of trauma, tenderness over the piriformis, pain relief with traction, and gluteal atrophy [8]. Still, current literature acknowledges a broader spectrum of presentations under the term "piriformis syndrome," including deep gluteal syndrome. Studies suggest that PS may account for up to 17.2% of low back pain cases, yet many clinicians remain unaware of its diagnostic and therapeutic nuances [9,10]. First described by Yeoman in 1928, the syndrome continues to raise important questions regarding its role in chronic sciatica [8]. Traditionally, sciatica was attributed mainly to mechanical compression of nerve roots, particularly from disc herniation. However, newer insights highlight the role of inflammation and immune-mediated nerve injury in radicular pain. Intriguingly, up to 76% of asymptomatic individuals may show disc herniation on MRI, and not all patients with sciatica have identifiable disc pathology [11]. These findings emphasize the need to explore non-discogenic, inflammatory, or soft tissue-related causes of sciatica-like symptoms.

Axial spondyloarthritis (axSpA) affects approximately 1% of the US population and is a significant cause of chronic inflammatory back pain (IBP). It typically presents before the age of 45 and may precede diagnosis by several years [12]. The 2009 Assessment of Spondyloarthritis International Society (ASAS) classification criteria enabled early diagnosis of non-radiographic axSpA (nr-axSpA) by incorporating MRI findings, human leucocyte antigen (HLA)-B27 status, and clinical features in the absence of radiographic sacroiliitis [13]. These patients often show less structural damage but share similar disease burden and inflammation with radiographic axSpA. Differentiating axSpA from degenerative conditions, such as intervertebral disc disease (IDD), spinal stenosis, and facet joint degeneration, requires a combination of clinical suspicion, imaging, and genetic testing [14].

The challenge in evaluating low back pain lies in its heterogeneous etiology. While much of it is categorized as nonspecific, a significant subset has inflammatory origins. Conditions like PS, sacroiliitis, and axSpA may all present with overlapping features, particularly buttock pain, which is frequently misattributed. Historically, sacroiliitis has been an underrecognized cause of sciatica. Emerging research proposes that inflammation, not just mechanical compression, can trigger radicular symptoms. These findings have led to interest in immunomodulatory treatments, including tumor necrosis factor (TNF) inhibitors, for cases where inflammation plays a central role [11].

We aimed to highlight a specific form of pseudo-sciatica of non-discogenic, extra-spinal origin, particularly related to PS and deep gluteal syndrome, that may overlap with spondyloarthritis features. Notably, PS-like presentations may fulfill ASAS or Amor classification criteria for spondyloarthritis, especially when buttock pain and sacroiliitis coexist.

We propose the term “Rafe’s sciatica” to describe these cases, where classic PS symptoms, when assessed thoroughly, align with spondyloarthritis features. Recognizing Rafe’s sciatica may improve early identification of axSpA, especially in patients presenting with buttock-originating radicular pain.

Study objective

The objective of this study was to investigate the association between PS and inflammatory disorders, particularly within the context of spondyloarthritis (SpA). Using the ASAS and Amor criteria, the study sought to evaluate whether PS-related presentations with buttock pain may warrant inclusion as diagnostic features of SpA. Specifically, this study was to identify and characterize Rafe’s sciatica as a novel, non-discogenic sciatica subtype associated with SpA, and to explore its diagnostic and clinical implications. By analyzing clinical cases with overlapping symptoms, we proposed and explored the clinical validity of “Rafe’s sciatica” as a new subtype of pseudo-sciatica within the SpA spectrum.

## Materials and methods

Study design

Our prospective case series was conducted to explore the association between SpA and sciatica through clinical, radiological, and biochemical observations, focusing on a new diagnostic approach, known as "Rafe’s sciatica." When participants underwent clinical assessment, data were collected in real-time to understand the clinical features and diagnostic criteria of SpA associated with sciatica. Ethical approval for the study was obtained from the Institutional Review Board of the Government Employees’ Hospital (Ref. No. SKH/IRB/2022/02).

Study setting and period

The study was conducted in the Department of Physical Medicine and Rehabilitation at the Government Employees’ Hospital in Dhaka, Bangladesh, from November 1, 2022, to July 19, 2023.

Participants

The study included 41 patients with sciatica who met the defined criteria. Patients presenting to the Department of Physical Medicine and Rehabilitation with buttock pain were screened for eligibility based on predefined criteria, reporting buttock pain from the sacrum to the greater trochanter, tenderness over the piriformis muscle, and symptom worsening during hip flexion, adduction, and internal rotation with or without sciatica.

Recruitment approach

Participants were recruited using a purposive sampling technique. No blinding procedures were applied during recruitment or data collection due to the nature of the study. Informed consent was obtained from all participants prior to inclusion.

Inclusion and exclusion criteria

**Figure 1:**
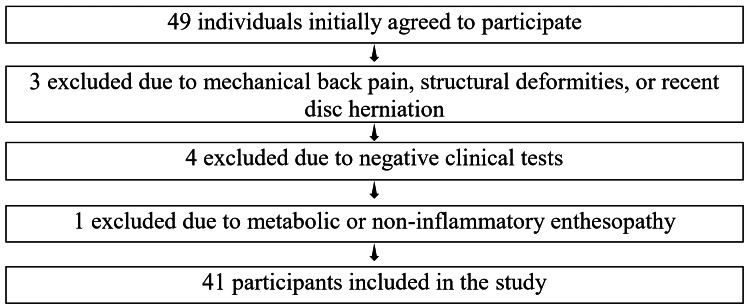
Inclusion and exclusion criteria.

Initially, 49 individuals agreed to participate in the study. After applying the inclusion and exclusion criteria, 41 participants were included in the final analysis. The inclusion criteria consisted of individuals under 45 years of age (n = 41), presenting with low back pain lasting more than three months, predominantly characterized by alternating or unilateral buttock pain. The pain had an insidious onset, lasted more than 30 minutes, was associated with morning stiffness (MS), worsened at night or with rest, and was typically relieved by nonsteroidal anti-inflammatory drugs (NSAIDs). Additionally, participants had a positive family history of SpA, psoriasis, psoriatic lesions, or arthritis associated with inflammatory bowel disease (IBD), and/or had sacroiliitis on X-ray or MRI, with some cases exhibiting elevated C-reactive protein (CRP)/erythrocyte sedimentation rate (ESR) levels (Figure 1).

Exclusion criteria included cases with mechanical back pain (n = 3) and structural deformities such as asymmetry, kyphosis, scoliosis, lordosis, spondylolisthesis, or recent herniated discs (n = 3), as assessed through clinical examinations like Adam's forward bend test, straight leg raise (SLR) test, and Schober’s test (Figure 1).

To investigate inflammatory enthesitis, we conducted the Achilles tendon squeeze test, the pressure pain test on the plantar fascia, and the costochondral joint compression test. We used ultrasound, which revealed hypoechoic (dark) areas at the entheses. Metabolic enthesopathy was excluded through serum tests for uric acid, calcium, and phosphate. For sacroiliitis detection, we followed the New York criteria for X-ray grading and utilized MRI to identify bone marrow edema on STIR (short tau inversion recovery)/T2-weighted images, as well as subchondral sclerosis on T1-weighted images, and synovitis, joint effusion, or capsulitis, indicative of inflammatory changes. Ultimately, 41 participants were included in the study after screening.

Data collection

**Figure 2:**
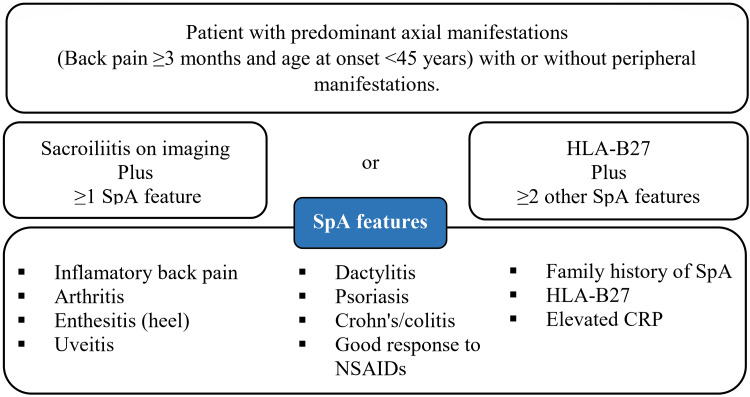
Assessment of Spondyloarthritis International Society (ASAS) classification criteria for spondyloarthritis. Source: Adapted from Ritchlin and Adamopoulos [13], with permission from the author and the publisher (BMJ Publishing Group Ltd.). HLA-B27: human leukocyte antigen B27; CRP: C-reactive protein; NSAIDs: nonsteroidal anti-inflammatory drugs; SpA: spondyloarthritis.

Data were gathered through medical record reviews and structured patient interviews. Demographic information, including age, gender, marital status, education, occupation, income, and residence, was recorded. Diagnostic evaluations used the ASAS (Figure 2) [13] and Amor criteria to assess SpA. Clinical history included duration of low back pain, morning stiffness, family history of SpA, psoriasis, uveitis, or IBD, and prior response to NSAIDs. Key findings included buttock pain, tenderness on palpation, positive piriformis stretch, FAIR (flexion, adduction, and internal rotation) and modified FAIR tests, and negative SLR and femoral nerve stretch (FNS) tests, though these negatives were not absolute for ruling out piriformis syndrome clinically. Functional assessment included numbness, paresthesia, and difficulty with walking or other activities. Laboratory and imaging data were also collected, including HLA-B27, rheumatoid factor (RF), anti-CCP antibodies, CRP/ESR, and MRI of the lumbar/sacroiliac spine to exclude spinal or disc pathology, as well as evaluation for enthesitis, dactylitis, and peripheral arthritis.

Imaging protocols

MRI of the sacroiliac joints and piriformis muscle was performed using spin-echo T1-weighted sequences in axial, sagittal, and coronal planes; fat-suppressed T2-weighted sequences in axial, sagittal, and coronal planes; and STIR sequences in the coronal plane.

Piriformis syndrome diagnosis

Piriformis syndrome in this study was diagnosed using a standardized clinical protocol. Patients presented with buttock pain radiating to the posterior thigh, aggravated by sitting or hip movements. Diagnosis was based on positive findings in the FAIR test (sensitivity is 88.1% and specificity is 83.2%) and the seated piriformis stretch test (sensitivity is 52% and high specificity is 90%), along with localized tenderness on palpation of the piriformis muscle [15,16]. In some cases, we injected local anesthetics (2% lidocaine) into the piriformis with ultrasound guidance. Improvement of pain strongly supports piriformis syndrome. To exclude differential diagnoses clinically, the SLR test and FNS tests were performed, both of which were consistently negative. MRI was employed to rule out lumbar pathology and to confirm the presence of sacroiliitis. An MRI fat suppression view or plain X-ray modified Ferguson view was applied. STIR and axial T2-weighted images also helped to clarify about targeted piriformis muscle pathology.

Assessment of enthesitis and sacroiliitis detection

To assess inflammatory enthesitis, the following tests were conducted: Achilles tendon squeeze test, pressure pain test on the plantar fascia, and costochondral joint compression test. Additionally, ultrasound imaging was performed to detect hypoechoic (dark) areas at entheses. Metabolic enthesopathy was ruled out using serum uric acid, calcium, and phosphate tests. For sacroiliitis detection, X-rays were evaluated based on the New York criteria grading system, while MRI scans were analyzed for bone marrow edema on STIR/T2-weighted images in the subchondral bone, subchondral sclerosis on T1-weighted images, and signs of inflammation such as synovitis, joint effusion, or capsulitis.

## Results

This study included 41 patients presenting with sciatica, all of whom fulfilled the 2010 Amor and ASAS criteria based on HLA-B27 positivity or radiological evidence of sacroiliitis on X-ray or MRI. The mean age of the participants was 39 years, with female predominance. A majority of the participants (24, 58.5%) were aged 45 years or younger, while the remaining 17 (41.5%) were >45 years of age. In terms of occupation, the majority were housewives (22, 53.7%), followed by 12 (29.3%) in service. Educational qualifications varied, with eight (19.5%) having no formal education, whereas 18 (43.9%) had completed secondary level schooling. Nearly half of the participants described themselves as rich (Table 1). Chronic low back pain lasting more than three months was reported by the vast majority (38, 92.7%). HLA-B27 was positive in 12 (29.3%) patients and negative in 29 (70.7%) patients. All the patients underwent MRI of the lumbosacral spine to rule out discogenic or spinal pathology inconsistent with their clinical presentation. Morning stiffness (MS) and alternating buttock pain (ABP) were reported by 35 (85.4%) of participants, with 28 (68.3%) experiencing stiffness lasting more than 30 minutes. While psoriasis was absent in all cases, uveitis was observed in three (7.3%) patients. A positive family history of spondyloarthritis (FH_SpA) was noted in 28 (68.3%) individuals. About 35 (85.4%) of participants reported persistent and radiating low back pain (PRLBP) and sacroiliac joint tenderness (SJT). Notably, all participants tested positive on the piriformis stretch test, reported numbness or paraesthesia, and had a positive FAIR and FADIR (flexion, adduction, internal rotation) test, indicating a diagnosis of piriformis syndrome. Peripheral arthritis was noted in nine (22.0%) cases, while 21 (51.2%) had a prior history of dactylitis. Enthesitis was found in 33 (80.5%) patients.

**Table 1:** Demographics, clinical presentation, laboratory findings, duration, and sacroiliitis on MRI or X-ray of 41 patients. P: positive; N: negative; DLBP: duration of low back pain; SJT: sacroiliac joint tenderness; PST: piriformis stretch test; Sac_MRI: sacroiliitis on MRI or X-ray; MS: morning stiffness; PA: peripheral arthritis; FH_SpA: family history of SpA; RA: rheumatoid arthritis; ABP: alternating buttock pain; Dac: dactylitis; Num or Para: numbness/paraesthesia; Ent: enthesitis; PRLBP: persistent and radiating low back pain; IBD: inflammatory bowel disease; HLA-B27: human leucocyte antigen B27; FNS: femoral nerve stretch test; SLR: straight leg raise test; FAIR: flexion, adduction, and internal rotation test.

Case series	Demographic status						DLBP (months)	Clinical presentation											Laboratory findings								Sac_MRI
	Gender	Age	Level of income	Marital status	Occupation	Level of education		PA	Dac	Ent	Numb or Para	PRLBP	ABP	MS (minutes)	Psoriasis	Uveitis	IBD	FH_spa	HLA-B27	RA	Anti-CCP	FNS	SLR	PST	FAIR	SJT	
Case-1	Female	17	Rich	Unmarried	Student	Secondary	<1	No	No	Yes	Yes	Yes	Yes	<30	No	No	No	Yes	Negative	N	N	N	N	P	P	P	Present
Case-2	Male	19	Poor	Unmarried	Student	Secondary	>3	No	No	No	Yes	Yes	Yes	>30	No	No	No	Yes	Positive	N	N	N	N	P	P	P	Present
Case-3	Male	23	Middle	Married	Service	Graduate	>3	No	Yes	Yes	Yes	Yes	Yes	<30	No	No	No	Yes	Positive	N	N	N	N	P	P	N	Present
Case-4	Female	26	Rich	Married	Service	Graduate	>3	No	Yes	Yes	Yes	Yes	No	0	No	No	No	No	Positive	N	P	N	N	P	P	N	Present
Case-5	Male	26	Rich	Unmarried	Service	Graduate	>3	No	Yes	No	Yes	Yes	No	<30	No	Yes	No	Yes	Positive	N	N	N	N	P	P	P	Present
Case-6	Male	30	Rich	Married	Driver	Higher secondary	>3	No	Yes	No	Yes	No	No	0	No	No	No	No	Negative	N	N	N	N	P	P	N	Absent
Case-7	Female	30	Rich	Married	Service	Secondary	>3	Yes	No	Yes	Yes	Yes	Yes	>30	No	No	No	No	Negative	N	N	N	N	P	P	P	Present
Case-8	Female	32	Middle	Married	House wife	Secondary	>3	Yes	No	Yes	Yes	Yes	Yes	>30	No	No	No	No	Positive	N	N	N	N	P	P	P	Present
Case-9	Male	33	Middle	Married	Service	Graduate	>3	No	No	Yes	Yes	Yes	No	>30	No	No	No	Yes	Negative	N	N	N	P	P	P	P	Present
Case-10	Female	38	Rich	Married	House wife	Secondary	>3	Yes	No	Yes	Yes	Yes	Yes	0	No	No	No	Yes	Negative	N	N	N	N	P	P	P	Present
Case-11	Female	43	Rich	Married	House wife	Higher secondary	>3	Yes	Yes	Yes	Yes	Yes	Yes	>30	No	Yes	No	Yes	Positive	N	N	N	N	P	P	P	Present
Case-12	Male	45	Middle	Married	Farmer	Illiterate	>3	Yes	Yes	Yes	Yes	Yes	Yes	>30	No	No	No	No	Negative	P	N	N	N	P	P	P	Present
Case-13	Female	46	Poor	Married	House wife	Illiterate	>3	No	Yes	Yes	Yes	Yes	Yes	10	No	No	No	Yes	Positive	N	N	N	N	P	P	P	Absent
Case-14	Female	46	Middle	Married	Laborer	Illiterate	<1	No	Yes	No	Yes	No	Yes	10	No	No	No	Yes	Negative	N	N	N	N	P	P	N	Present
Case-15	Female	47	Middle	Married	House wife	Secondary	>3	No	Yes	Yes	Yes	Yes	Yes	0	No	No	No	Yes	Negative	N	N	N	N	P	P	P	Present
Case-16	Female	47	Rich	Married	House wife	Graduate	>3	No	Yes	Yes	Yes	Yes	Yes	>30	No	No	No	No	Negative	N	N	N	N	P	P	P	Present
Case-17	Female	47	Rich	Married	Service	Post graduate	>3	No	Yes	Yes	Yes	Yes	Yes	>30	No	No	No	No	Negative	N	N	N	N	P	P	P	Present
Case-18	Female	48	Poor	Married	House wife	Secondary	>3	No	Yes	Yes	Yes	No	Yes	>30	No	No	No	Yes	Negative	N	N	N	P	P	P	P	Present
Case-19	Female	51	Poor	Married	House wife	Illiterate	>3	No	No	Yes	Yes	Yes	Yes	>30	No	No	No	Yes	Negative	N	N	N	N	P	P	P	Present
Case-20	Male	52	Rich	Married	Service	Post graduate	>3	No	No	Yes	Yes	Yes	Yes	>30	No	No	No	No	Positive	P	N	N	N	P	P	P	Present
Case-21	Female	54	Poor	Widow	House wife	Secondary	>3	No	No	No	Yes	Yes	Yes	>30	No	No	No	Yes	Negative	N	N	N	N	P	P	P	Present
Case-22	Female	54	Poor	Married	House wife	Secondary	>3	No	No	Yes	Yes	Yes	Yes	>30	No	No	No	Yes	Negative	N	N	N	N	P	P	P	Absent
Case-23	Female	62	Rich	Married	House wife	Secondary	>3	No	No	Yes	Yes	Yes	Yes	>30	No	No	No	Yes	Negative	N	N	N	N	P	P	P	Present
Case-24	Male	31	Rich	Married	Service	Graduate	>3	Yes	Yes	No	Yes	No	No	0	No	No	No	Yes	Negative	N	N	N	N	P	P	N	Absent
Case-25	Female	32	Middle	Married	House wife	Higher secondary	>3	Yes	No	Yes	Yes	Yes	Yes	>30	No	No	No	Yes	Negative	N	N	N	N	P	P	P	Present
Case-26	Female	34	Middle	Married	House wife	Secondary	>3	No	No	Yes	Yes	Yes	Yes	>30	No	No	No	Yes	Positive	N	N	N	N	P	P	P	Present
Case-27	Female	35	Rich	Married	House wife	Secondary	>3	Yes	No	Yes	Yes	Yes	No	>30	No	No	No	No	Negative	N	N	N	P	P	P	P	Present
Case-28	Female	37	Rich	Married	House wife	Graduate	>3	Yes	No	Yes	Yes	Yes	Yes	0	No	No	No	Yes	Negative	N	N	N	N	P	P	P	Present
Case-29	Female	44	Middle	Married	House wife	Secondary	>3	No	Yes	Yes	Yes	Yes	Yes	>30	No	Yes	No	Yes	Positive	N	N	N	N	P	P	P	Present
Case-30	Female	46	Poor	Married	House wife	Higher secondary	>3	No	Yes	Yes	Yes	Yes	Yes	>30	No	No	No	Yes	Negative	P	N	N	N	P	P	P	Present
Case-31	Female	49	Middle	Married	House wife	Illiterate	>3	No	Yes	Yes	Yes	Yes	Yes	10	No	No	No	No	Positive	N	N	N	N	P	P	P	Absent
Case-32	Male	53	Middle	Married	Farmer	Illiterate	<1	No	Yes	No	Yes	No	Yes	10	No	No	No	No	Negative	N	N	N	N	P	P	N	Present
Case-33	Female	32	Rich	Married	House wife	Secondary	>3	No	Yes	Yes	Yes	Yes	Yes	>30	No	No	No	Yes	Negative	N	N	N	N	P	P	P	Present
Case-34	Female	33	Rich	Married	Service	Secondary	>3	No	Yes	Yes	Yes	Yes	Yes	>30	No	No	No	Yes	Negative	N	P	N	N	P	P	P	Present
Case-35	Female	52	Poor	Married	Service	Graduate	>3	No	Yes	Yes	Yes	Yes	Yes	>30	No	No	No	No	Negative	N	N	N	N	P	P	P	Present
Case-36	Female	23	Poor	Unmarried	Service	Secondary	>3	No	Yes	Yes	Yes	No	Yes	>30	No	No	No	Yes	Negative	N	N	N	P	P	P	P	Present
Case-37	Female	27	Rich	Married	House wife	Secondary	>3	No	No	Yes	Yes	Yes	Yes	>30	No	No	No	Yes	Negative	N	N	N	N	P	P	P	Present
Case-38	Female	31	Poor	Married	House wife	Illiterate	>3	No	No	Yes	Yes	Yes	Yes	>30	No	No	No	Yes	Positive	P	N	N	N	P	P	P	Present
Case-39	Female	64	Poor	Married	Service	Post graduate	>3	No	No	No	Yes	Yes	Yes	>30	No	No	No	No	Negative	N	N	N	N	P	P	P	Present
Case-40	Male	58	Rich	Married	Laborer	Illiterate	>3	No	No	Yes	Yes	Yes	Yes	>30	No	No	No	Yes	Negative	N	N	N	N	P	P	P	Absent
Case-41	Female	31	Middle	Married	House wife	Secondary	>3	No	No	Yes	Yes	Yes	Yes	>30	No	No	No	Yes	Negative	N	N	N	N	P	P	P	Present

Rheumatoid factor (RA) was negative in 37 (90.2%) cases, though anti-cyclic citrullinated peptide (anti-CCP) antibodies were negative in nearly all patients (39, 95.1%). None tested positive on the FNS test. Imaging confirmed sacroiliitis in 35 (85.4%) cases through either X-ray or MRI (Table 1).

**Figure 3:**
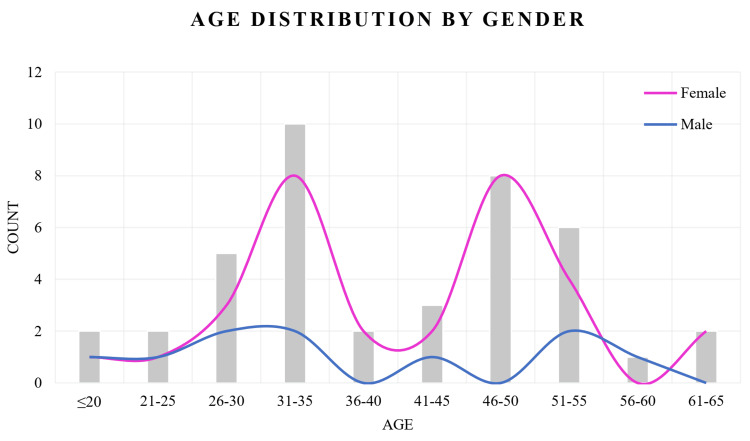
Age distribution by gender.

Figure 3 illustrates the age distribution of the participants stratified by gender. The x-axis represents different age groups, while the y-axis indicates the number of individuals within each category. Among the females, the highest number was observed in the 31-35 and 46-50 year age groups, each comprising eight (25.81%) participants. In contrast, male participants were fewer in number and showed a more evenly distributed pattern, with minor peaks noted in the 26-30, 31-35, and 51-55 year age groups. Notably, males were absent in the 36-40, 46-50, and 61-65 year age categories, whereas females were represented across all age groups (Figure 3).

**Figure 4:**
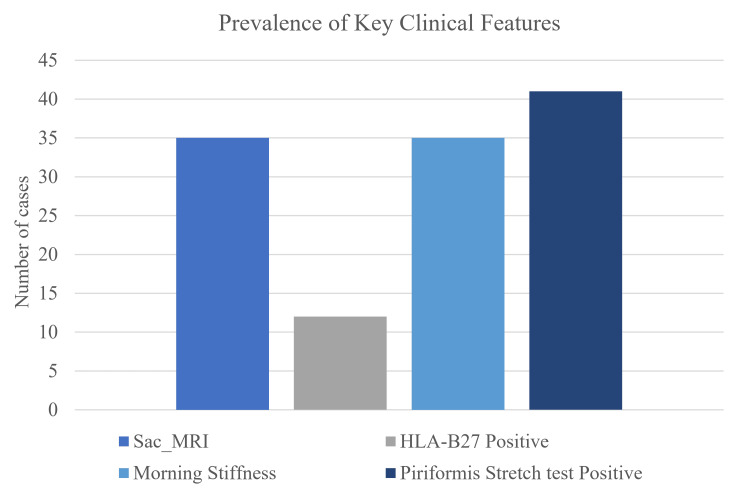
Prevalence of key clinical features. Sac_MRI: sacroiliitis on MRI or X-ray; HLA-B27: human leukocyte antigen B27.

Figure 4 presents a bar chart illustrating the prevalence of key clinical characteristics among the study participants. Sacroiliitis on MRI was identified in 35 patients (85.37%), indicating a high prevalence of axial involvement. HLA-B27 positivity was observed in 12 cases (29.27%), suggesting a potential genetic predisposition in a subset of individuals presenting with Rafe’s sciatica. Additionally, morning stiffness was reported by 35 participants (85.37%), further supporting the inflammatory nature of the condition and helping to distinguish it from mechanical causes of sciatica (Figure 4).

**Figure 5:**
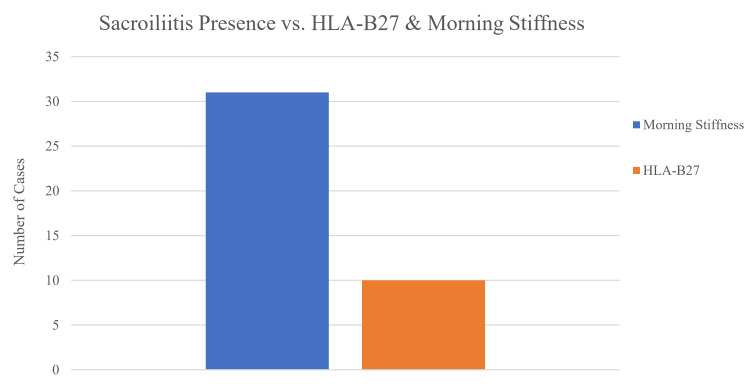
Sacroiliitis presence vs. HLA-B27 and morning stiffness. HLA-B27: human leukocyte antigen B27.

Figure 5 illustrates the distribution of HLA-B27 positivity and the presence of morning stiffness among individuals with confirmed sacroiliitis. Among these patients, morning stiffness was reported in 31 cases (75.61%), whereas only 10 patients (24.39%) tested positive for the HLA-B27 marker (Figure 5).

**Figure 6:**
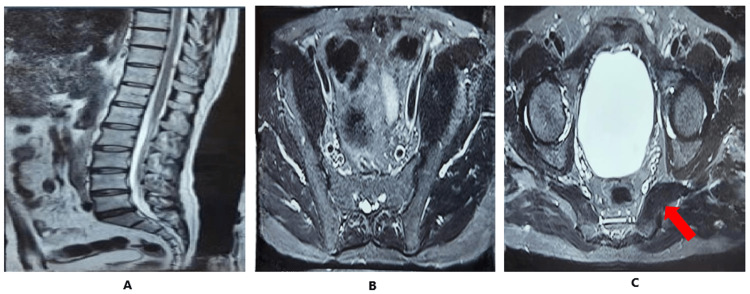
MRI findings suggestive of right-sided sacroiliitis and involvement of right piriformis muscle. (A) Sagittal T2-weighted images of the lumbosacral spine, having no significant discomfort pathology, revealed hypointense areas in the sacrum and ilium adjacent to the right sacroiliac joint, indicating marrow changes. (B) Corresponding coronal STIR (short tau inversion recovery) images demonstrated heterogeneous hyperintense signals at the same sites, consistent with active bone marrow edema and inflammation. (C) STIR and axial T2-weighted images further illustrated that the right piriformis muscle appears mildly hypertrophied in diameter compared to the left.

The MRI findings were suggestive of right-sided sacroiliitis with concurrent involvement of the right piriformis muscle. On sagittal T2-weighted images of the lumbosacral spine, there was no evidence of significant spinal pathology; however, hypointense signal changes were noted in the sacrum and ilium adjacent to the right sacroiliac joint, indicating underlying marrow changes. Coronal STIR images revealed heterogeneous hyperintense signals in the same regions, consistent with active bone marrow edema and inflammatory changes characteristic of sacroiliitis. Additionally, axial STIR and T2-weighted sequences demonstrated that the right piriformis muscle appears mildly hypertrophied when compared to the contralateral side, raising the possibility of secondary myositis or reactive muscular changes associated with the adjacent inflammatory process (Figure 6).

## Discussion

Sciatica is a common issue in clinical practice, generally attributed to nerve root compression, often related to disc herniation, foraminal stenosis, or spondylolisthesis. However, "sciatica" itself lacks a consistent definition in literature, commonly describing symptoms rather than a diagnosis. Although herniated lumbar discs are a frequent cause, alternative pathologies, such as inflammatory processes, may also play a role. Clinicians often use "sciatica" interchangeably with "radicular pain," a practice that may risk diagnostic oversights [1]. An understanding of deep gluteal anatomy and its interaction with symptomatology is essential for correct sciatica-related diagnoses and management. Physical examination is foundational in this, though it has limitations, and imaging studies can sometimes fail to confirm sciatica where inflammation is the root cause. This can complicate the accurate diagnosis of sacroiliac joint pathology, demanding a nuanced clinical approach [3]. A number of sciatica patients may actually experience IBP due to SpA. axSpA is a key cause of chronic low back pain with an inflammatory profile. Due to subtle clinical features and limited radiographic findings in the initial stages, early diagnosis of axSpA is challenging, particularly in primary care. However, HLA-B27 testing and MRI have enhanced the ability to identify axSpA, helping to differentiate it from other conditions like disc herniation or spinal stenosis [12]. Some reports show that sacroiliac joint dysfunction can radiate pain to the leg, creating symptoms similar to sciatica. This referral pattern likely results from shared innervation between the sacroiliac joint and sciatic nerve, especially involving the L5-S4 nerve roots. Cytokine release may be triggered by inflammation in the sacroiliac joint, which can irritate surrounding neural structures, thus mimicking radicular symptoms. MRI may reveal sacroiliac capsulitis or enthesitis, which can contribute to sciatica-like pain [17]. Research has highlighted the role of the piriformis muscle in sciatica. PS occurs when the piriformis muscle compresses the sciatic nerve, resulting in buttock and thigh pain. There is a notable overlap between PS and sacroiliitis, as both can lead to leg pain. Sacroiliitis is traditionally associated with SpA, although PS may coexist with SpA or act independently, complicating the differential diagnosis. The interplay between the piriformis muscle, sciatic nerve, and sacroiliac joint broadens the concept of PS, including cases where piriformis inflammation leads to sciatic nerve entrapment [8].

We aimed to present a logical discussion on the generation of piriformis syndrome, with or without sacroiliitis, in SpA cases. Here, every patient was suspected of SpA according to the ASAS criteria and also fulfilled the Amor criteria. The pathogenesis of PS is not believed to be significantly impacted by anatomical variations of the sciatic nerve and piriformis muscle. Our best estimate is that dynamic sciatic nerve entrapment at the infra-piriformis fossa is a common occurrence; however, a new description of the piriformis musculotendinous junction has revealed a common variant [18]. Here, we also want to address Rafe’s sciatica, which is associated with piriformis syndrome, as well as capsulitis, synovitis, and enthesopathy due to its developmental issue. The piriformis muscle originates from several anatomical locations. The piriformis muscle has multiple points of origin: the anterior sacral foramina, the sacroiliac joint capsule, the gluteal surface of the ilium (near the posterior inferior iliac spine), and even the sacrotuberous ligament (more precisely, the superior part of the pelvic surface). It also comes from the middle three pieces of the sacrum and the adjoining lateral mass, as well as from the superior margin of the greater sciatic notch and the ilium's gluteal surface [19]. The interosseous sacroiliac ligament primarily connects the sacrum and ilium. To prevent the joint from nutation, the sacrotuberous ligament works in conjunction with the posterior sacroiliac ligament. The sacrospinous ligament is followed by the sacrotuberous ligament. There are usually more receptors that are sensitive to pain than mechanoreceptors in the sacroiliac joint. The predominant neural branches to the piriformis originate from the superior gluteal nerve (70%), which emerges from the dorsal divisions of the L4, L5, and S1 nerve roots of the sacral plexus and traverses above it, along with the ventral rami of S1 and S2 [20].

According to the ASAS handbook (2009), MRI may reveal capsulitis, synovitis, and enthesitis, with capsulitis being analogous to synovitis, typically affecting the anterior and posterior capsule of the sacroiliac joint. The anterior aspect of the joint capsule progressively extends into the periosteum of the iliac and sacral bones, so producing an enthesis. Capsulitis may expand significantly medially and laterally into the periosteum. Hyperintense signal is observed on STIR images and/or contrast-enhanced T1-weighted fat-saturated imaging at locations where ligaments and tendons anchor to bone, including the retroarticular space (interosseous ligaments). The signal may propagate to bone marrow and soft tissue [21]. Ultrasonographic findings of the piriformis in the affected instances further corroborate this. Researchers determined that the average ultrasound-guided piriformis muscle (PM) thickness was greater in PS subjects than in healthy subjects, with mean thicknesses of 1.16 ± 0.13 cm and 0.85 ± 0.11 cm, respectively (p < 0.05) [22].

Bone marrow edema (BME) and fat lesions were most pronounced in patients with axial SpA but also occurred in other groups, particularly women with postpartum buttock/pelvic pain [23]. So, the involvement of the periarticular area can be more pronounced in axial SpA. Imaging studies from the paravertebral region to the sciatic notch were unremarkable, but an MRI of the piriformis muscle showed an enlargement of the left muscle with an enlargement and a slight anterior displacement of the left sciatic nerve, having no lumbosacral disk abnormalities [24].

Rafe’s sciatica is also supported by the Berlin criteria for chronic low back pain, having buttock pain, indicating the nature of that inflammatory type [25]. Like awakening in the second half of the night because of back pain and alternating buttock pain. The European Spondyloarthropathy Study Group criteria may be correlated with Rafe’s syndrome, inflammatory spinal pain/synovitis predominantly in the lower limb, with any one among (a) sacroiliitis, (b) buttock pain alternating between right and left gluteal area, and (c) enthesopathy [17]. Therefore, the pathophysiology of Rafe’s sciatica and its association with capsulitis, synovitis, and enthesopathy may be supported by both anatomy and scientific evidence.

Wong et al. also noticed two cases of pseudo sciatica with sacroiliitis cases in 2005, which support our statement [11]. The first person, a 28-year-old, had left-sided sacroiliitis with initially left-sided sciatica, followed by classical right-sided sciatica after 15 months, having right-sided sacroiliitis, which we want to say is alternate buttock pain with piriformis syndrome. Here, the patient fulfilled the ASAS criteria for axial SpA. The second case, a 29-year-old man diagnosed with psoriatic arthritis, having bilateral sacroiliitis, came with classical sciatica, which we want to take into account as Rafe’s sciatica.

An interesting finding was found in another study of 74 patients (63.8%) who exhibited pathologies related to PM. Primary causes were detected in 12, and secondary causes in 62 patients. PM enlargement was found in 45.9% of patients, abnormal PM signal intensity/density in 40.5%, and sciatic neuritis in 25.7%. Imaging revealed an unknown underlying medical condition and altered treatment planning. Secondary PM etiologies appear to prevail. In suspected PMs, PM enlargement represented the most common imaging finding [26].

Wong et al. noticed how sacroiliitis can lead to sciatic symptoms in two primary ways: through referred pain and the release of inflammatory mediators that affect nearby nerves [11]. Many patients describe pain just below the posterior superior iliac spine, but some find it radiating further down their legs. Their research found that about one-third of sacroiliac joint injections revealed a surprising connection to surrounding nerve structures, highlighting a direct anatomical link that can lead to radicular pain. Interestingly, in their cases, even though the straight leg raising test indicated some irritation of the sciatic nerve, the patients showed no neurological deficits. This reinforces the idea that sacroiliitis should be a key consideration when patients present with sciatica. Unlike pain from a herniated disc, which often lingers during rest and activity, sacroiliac pain may have different characteristics. For accurate diagnosis, MRI is the go-to option, boasting a sensitivity of 95% compared to the lower rates of X-rays and scintigraphy. When it comes to treatment, sacroiliitis and disc herniation require different approaches. For patients who do not respond to standard care, corticosteroid injections may offer temporary relief, but this typically lasts only about three months. Additionally, high levels of TNF-alpha found in biopsies from the sacroiliac joint suggest that biologic therapies, such as infliximab and etanercept, can significantly improve the quality of life for those with ankylosing spondylitis. Therefore, understanding sacroiliitis as a potential source of leg pain is crucial for providing the right diagnosis and treatment options [11].

Two subtypes of axSpA have been identified: classical ankylosing spondylitis (AS), which is defined by pre-existing structural alterations in the sacroiliac joints, and so-called nr-axSpA, in which these alterations are not present. The ASAS criteria for axSpA classification, which are used to make this differentiation, are not, however, diagnostically appropriate. So, should Rafe’s sciatica take this into account to be considered evidence of nr-axSpA? Moreover, this study invites further research to explore additional presentations of SpA, potentially discovering new insights into pathophysiology and aiding in early diagnosis.

Limitations

Our study has several limitations that should be considered when interpreting the findings. First, the relatively small sample size may affect the statistical power and limit the generalizability of the results. Future research involving larger cohort studies is warranted to validate these findings. Second, the lack of a control group restricts direct comparison with other potential causes of sciatica, which is crucial for strengthening the diagnostic specificity of the observed patterns. Third, the potential for diagnostic overlap with related conditions represents a limitation that could have affected the accuracy of our findings. However, due to the small, uncontrolled case series design, causal inference is limited. Lastly, further investigations utilizing advanced imaging modalities, particularly MRI, may offer more detailed assessments of associated features such as capsulitis and enthesopathy, enhancing our understanding of their clinical relevance.

## Conclusions

Rafe’s sciatica expands the clinical spectrum of SpA by identifying piriformis inflammation as a relevant and underrecognized source of inflammatory sciatica. Considering this condition in SpA patients presenting with sciatic-like symptoms in the absence of disc pathology may enhance diagnostic precision. Integrating piriformis-related inflammation into SpA diagnostic standards could allow earlier and more accurate recognition, while individualized anti-inflammatory treatment approaches may improve outcomes and reduce misdiagnosis compared with mechanical or surgical management. Although our findings underscore its potential clinical significance, further validation through larger, comparative studies is required to refine diagnostic criteria and inform effective treatment strategies.
